# Chemical Starting Matter for HNF4α Ligand Discovery and Chemogenomics

**DOI:** 10.3390/ijms21217895

**Published:** 2020-10-24

**Authors:** Isabelle Meijer, Sabine Willems, Xiaomin Ni, Jan Heering, Apirat Chaikuad, Daniel Merk

**Affiliations:** 1 Institute of Pharmaceutical Chemistry, Goethe University Frankfurt, Max-von-Laue-Str. 9, 60438 Frankfurt, Germany; a01568041@unet.univie.ac.at (I.M.); willems@pharmchem.uni-frankfurt.de (S.W.); ni@em.uni-frankfurt.de (X.N.); chaikuad@pharmchem.uni-frankfurt.de (A.C.); 2 Structural Genomics Consortium, BMLS, Goethe-University Frankfurt, Max-von-Laue-Str. 15, 60438 Frankfurt, Germany; 3 Fraunhofer Institute for Molecular Biology and Applied Ecology IME, Branch for Translational Medicine and Pharmacology TMP, Theodor-Stern-Kai 7, 60596 Frankfurt, Germany; Jan.Heering@ime.fraunhofer.de

**Keywords:** Orphan nuclear receptor, hepatocyte nuclear factor 4α, MODY, type 2 diabetes, fragment-based design, drug discovery

## Abstract

Hepatocyte nuclear factor 4α (HNF4α) is a ligand-sensing transcription factor and presents as a potential drug target in metabolic diseases and cancer. In humans, mutations in the HNF4α gene cause maturity-onset diabetes of the young (MODY), and the elevated activity of this protein has been associated with gastrointestinal cancers. Despite the high therapeutic potential, available ligands and structure–activity relationship knowledge for this nuclear receptor are scarce. Here, we disclose a chemically diverse collection of orthogonally validated fragment-like activators as well as inverse agonists, which modulate HNF4α activity in a low micromolar range. These compounds demonstrate the druggability of HNF4α and thus provide a starting point for medicinal chemistry as well as an early tool for chemogenomics.

## 1. Introduction

The transcriptional regulator hepatocyte nuclear factor 4α (HNF4α, NR2A1) [1,2] belongs to the protein family of nuclear receptors which are ligand-sensing transcription factors. HNF4α acts as an obligate homodimer [3] and has constitutive transactivation activity [1]. It was initially considered as an orphan nuclear receptor [1,2] prior to the identification of linoleic acid as a potential endogenous ligand, whose binding, however, has been reported to hardly affect the receptor’s transcriptional activity [4]. HNF4α is mainly found in hepatocytes, enterocytes, and pancreatic β-cells [5,6], and exhibits key regulatory roles in intestine [7], liver [8], and pancreas [9]. Its dysregulation has been associated with gastrointestinal and metabolic diseases [5,10,11] as well as gastrointestinal cancers [5,12,13]. Mutations within the HNF4α gene cause the heritable form of type 2 diabetes, maturity-onset diabetes of the young 1 (MODY-1) [10,14,15,16], highlighting the receptor’s crucial role in metabolic homeostasis. Despite an attractive potential for pharmacological interventions in diabetes or cancer, the collection of ligands that can modulate the transcriptional activity of HNF4α is scarce. Kiselyuk et al. [5] have previously reported antagonists of HNF4α with low micromolar potencies that decreased the receptor levels as well as expression of the targeted genes in cellular settings. In addition, a series of naphthofuranes [17] was found to bind the HNF4α ligand-binding domain (LBD) and enhance the receptor’s transcriptional activity. While these important observations demonstrate the possibility of HNF4α modulation with small molecules, novel HNF4α ligands are needed to overcome the limited potency, physicochemical restrictions, and lack of chemical diversity of available HNF4α modulators.

To expand the collection of HNF4α ligands, we screened a collection of 480 drug fragments for HNF4α modulation in a cellular setting. Dose-response profiling, control experiments, effects on HNF4α-regulated gene expression in native cellular setting, and interaction studies with the recombinant HNF4α LBD fully confirmed a set of appealing molecules as HNF4α modulators including agonists and inverse agonists. The most active compounds modulated the orphan nuclear receptor with low micromolar potencies and modulated mRNA expression of the HNF4α-regulated gene fructose-1,6-bisphosphatase 1 (FBP1) in human hepatocytes. For three HNF4α modulators, isothermal titration confirmed direct binding to the recombinant HNF4α LBD with micromolar affinities. This set of chemically diverse HNF4α ligands will serve as a starting point for medicinal chemistry, enabling the development of tool compounds for further pharmacological studies on the role of HNF4α in diseases.

## 2. Results

### 2.1. Primary Screening

**Figure 1:**
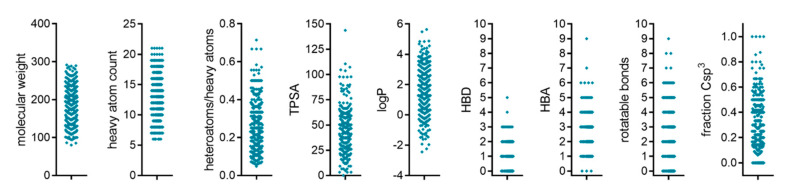
Characteristics and feature distribution of the fragment screening library. TPSA—topological polar surface area; HBD—H-bond donor; HBA—H-bond acceptor.

To search for new modulators of HNF4α activity without previous knowledge on the structure–activity relationship of HNF4α ligands, we screened a collection of 480 fragments derived from FDA approved drugs for their abilities to modulate HNF4α activity in vitro (the fragment structures contained in the library and associated primary screening data are provided as Table S1). This library was chemically diverse in terms of feature distribution (Figure 1) and scaffolds, providing an attractive unbiased and economic entry to HNF4α ligand discovery.

For the primary screening system, we employed a hybrid Gal4 reporter gene assay in transiently transfected HEK293T cells. Therein, a Gal4 responsive firefly luciferase construct served as a reporter gene to determine the transcriptional activity of a hybrid receptor composed of the human HNF4α LBD and the DNA-binding domain of Gal4 from yeast. A constitutively active (SV40 promoter) renilla luciferase construct was additionally present to monitor the transfection efficiency and toxicity of test compounds. This system is advantageous as it captures various characteristics of nuclear receptor modulators including potency, type of activity, efficacy, and cell permeability [18]. In accordance with previous reports on the behavior of HNF4α [1], the chimeric Gal4-HNF4α receptor revealed marked constitutive transcriptional activity in the absence of ligands.

**Figure 2:**
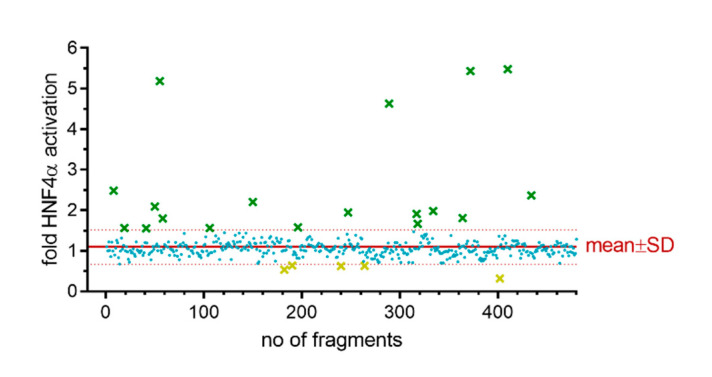
Activity distribution and primary hits in the fragment screening. Red line shows the mean, dotted red lines show mean ± SD. Each point represents the activity of one fragment at 50 µM expressed as mean fold activation vs. 0.2% DMSO from two biologically independent repeats. Green crosses refer to HNF4α agonist candidates, yellow crosses refer to inverse HNF4α agonist candidates, blue dots represent fragments without activity on Gal4-HNF4α.

The entire fragment library was tested for Gal4-HNF4α modulation using this primary screening assay, which was performed in two biologically independent repeats using compounds at 50 µM concentration. Reporter activity for the entire fragment set was narrowly distributed with a mean of 1.09 and a standard deviation of 0.43. Fragments exhibiting a fold activation outside the mean ± SD region were evaluated as primary hits. Eighteen fragments induced reporter activity to values above 1.52-fold activation (mean + SD) and were considered as potential agonists. Five compounds suppressed the reporter signal to values below 0.67-fold activation (mean - SD) and were considered as potential inverse agonists (Figure 2). The primary hits were then manually curated for toxic compounds (as observed by effects on constitutively active renilla luciferase) and undesired structures (PAINS), leaving a primary hit collection of eleven fragments (**1**-**11**) for validation (Table 1). This primary hit list comprised eight potential HNF4α activators (**1**–**8**) and three inverse agonist candidates (**9**–**11**).

### 2.2. Hit Validation

HNF4α modulation of the primary screening hits **1**–**11** was subsequently reproduced with fresh material in four biologically independent repeats in duplicates. For compounds exhibiting pronounced toxicity at 50 µM, concentration was reduced (**6**: 5 µM; **9**: 30 µM). Fragments **5** and **11** revealed a tendency for HNF4α modulation at 50 µM and were subsequently characterized at 100 µM. Additionally, the primary hits were further validated in control experiments involving the potent, ligand-independent transcriptional activator Gal4–VP16 to replace Gal4–HNF4α with otherwise identical assay settings and test compound concentrations (Table 1). Like Gal4–HNF4α, Gal4–VP16 has high constitutive transcriptional inducer activity but is not responsive to ligands. Thus, fragments affecting reporter activity in the Gal4–VP16 setting likely modulate non-specific cellular processes. Fragments that exhibited HNF4α modulation in this initial follow-up and had no effects on Gal4–VP16-induced reporter activity were further studied by full dose-response characterization. **1**–**3**, **8**, and **11** showed no statistically significant activity in the Gal4–HNF4α assay compared to the Gal4–VP16 control, suggesting unspecific effects. Fragments **4**–**7** were confirmed as HNF4α agonists and fragments **9** and **10** exhibited preliminarily validated inverse HNF4α agonism.

**Figure 3:**
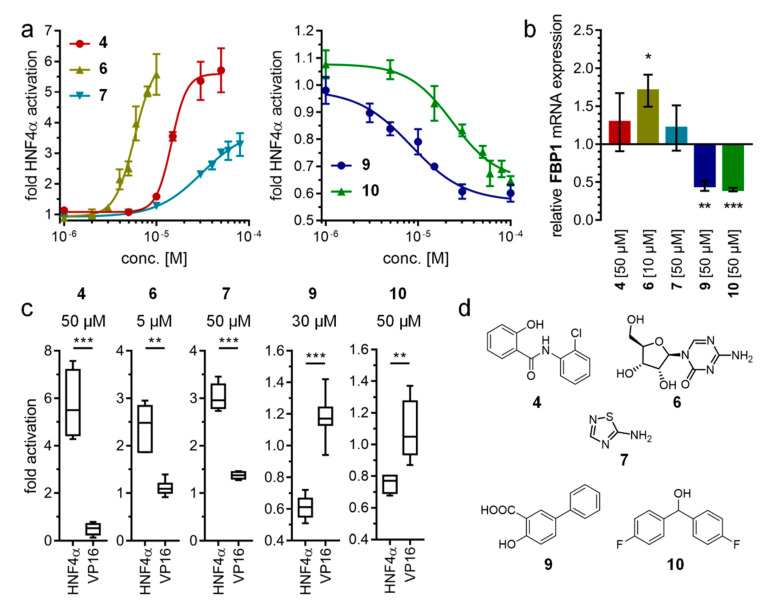
In vitro characterization of HNF4α modulators **4**, **6**, **7**, **9**, and **10**. (**a**) Dose–response curves of **4**, **6**, **7**, **9**, and **10** on Gal4-HNF4α (mean ± S.E.M.; *n* ≥ 4). (**b**) Effects of **4**, **6**, **7**, **9**, and **10** on mRNA expression of fructose-1,6-bisphosphatase 1 (FBP1) in human hepatocytes (HepG2). HNF4α activators **4**, **6**, and **7** promoted FBP1 expression, while the inverse HNF4α agonists **9** and **10** decreased FBP1 mRNA levels. Data are the mean ± S.E.M. mRNA levels determined by qRT-PCR and analyzed by the 2^−ΔΔCt^ method; *n* = 3. (**c**) Control experiments on Gal4-VP16 (boxplots show mean, min.-max.; *n* ≥ 4). (**d**) Chemical structures of **4**, **6**, **7**, **9**, and **10**. * *p* < 0.05, ** *p* < 0.01, *** *p* < 0.001 (*t*-test).

Dose-response profiling (Table 2, Figure 3) revealed **6** as the strongest and most potent HNFα agonist with an EC_50_ value of 5.8 µM and a high 6.1-fold maximum activation efficacy. Fragments **4** (EC_50_ = 15 µM, 5.6-fold act.) and **7** (EC_50_ = 31 µM, 3.8-fold act.) comprised slightly weaker agonist potencies than **6**, while fragment **5** (EC_50_ > 100 µM) was considerably less active. The inverse agonists **9** (IC_50_ = 8 µM) and **10** (IC_50_ = 24 µM) demonstrated low micromolar activity, and both diminished remaining HNF4α activity by approximately half.

To capture HNF4α modulation by **4**, **6**, **7**, **9**, and **10** in a more physiological setting, we treated HNF4α-expressing [19] human hepatocytes (HepG2) with the HNF4α modulators and then determined the mRNA expression levels of the HNF4α-regulated gene fructose-1,6-bisphosphatase 1 (FBP1) [19] by quantitative real-time PCR (qRT-PCR). In accordance with its activity in the Gal4-HNF4α assay, the most active HNF4α activator **6** significantly promoted FBP1 expression in HepG2 cells. The less potent agonists **4** and **7** revealed a tendency to enhanced FBP1 mRNA levels. The inverse HNF4α agonists **9** and **10** robustly decreased FBP1 expression, confirming their activity observed in the screening system as well. These results further validate cellular HNF4α modulation by **4**, **6**, **7**, **9**, and **10** and demonstrate that these compounds can also be used as initial tools to study HNF4α function in native settings.

For a preliminary quality assessment of this validated HNF4α ligand collection as a starting matter for medicinal chemistry, we calculated key physicochemical features and ligand-efficiency metrics [20,21] (Table 3). As feasibly expected from their chemical structures, the HNF4α agonists **6** and **7** comprised favorably low lipophilicity with low predicted logP values. Fragment **7** possessed the highest ligand efficiency (LE) owing to its small size but also a preferable size-independent ligand efficiency (SILE). Fragment **6**, due to its exceptional polarity, was superior regarding lipophilic ligand efficiency (LLE). Both inverse HNF4α agonists **9** and **10** revealed acceptable lipophilicity with predicted logP values of 3–4. LE, LLE, and SILE were comparable for both compounds and favorably high for fragment-like screening hits.

**Figure 4:**
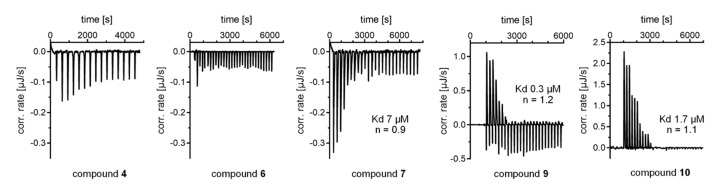
Isothermal titration calorimetry (ITC) demonstrated binding of **7**, **9**, and **10** to the recombinant HNF4α LBD protein, confirming their HNF4α-mediated activity. Recombinant HNF4α LBD protein (10–20 µM) was titrated with ligands (50–200 µM).

To orthogonally confirm direct interaction between HNF4α and the identified hits, we determined the binding of **4**, **6**, **7**, **9**, and **10** to the recombinant HNF4α LBD protein by isothermal titration calorimetry (ITC). In line with the nuclear receptor’s mode of action [3], we observed dimerization of the HNF4α LBD protein and dimer dissociation upon dilution, which hindered inverse ITC experiments. Fragments **4**, **6**, **7**, **9**, and **10** were, therefore, titrated to the HNF4α LBD (Figure 4). Binding of **5** was not studied due to its weak potency observed in the cell-based assay. The ITC results indicated very weak HNF4α binding of **4** and revealed no proper binding isotherm for **6**. According to this observation, the effects of **6** on HNF4α activity in the Gal4-HNF4α assay and in HepG2 cells seem to be mediated by indirect pathways and might, for example, involve HNF4α activation by phosphorylation. Since the control experiments on Gal4-VP16 revealed no unspecific effects of **6** and since **6** caused marked upregulation of the HNF4α-regulated FBP1 in hepatocytes, its activity still appears HNF4α-mediated. Current evidence, however, does not support direct HNF4α agonism of **6**, and further studies are needed to elucidate the mechanism by which the compound promotes HNF4α activity. For fragments **7**, **9**, and **10**, ITC experiments clearly demonstrated their binding with micromolar binding affinities (**7**: K_d_ ~7 µM; **9**: K_d_ ~0.3 µM; **10**: K_d_ ~1.7 µM), orthogonally validating their direct effect on HNF4α modulation. In addition, fragment **4** likely activated the nuclear receptor through interaction with the LBD despite weak binding affinity and **5** was another weak HNF4α activator according to the cellular experiments.

## 3. Discussion

Our systematic screening yielded a considerable hit-rate, leading to the discovery of three fully validated HNF4α ligands (**7**, **9**, **10**) exhibiting low micromolar potencies and binding affinities as well as two additional weaker HNF4α modulators (**4**, **5**). These hits were highly chemically diverse. We observed no preference for certain chemotypes or functional groups and, surprisingly, only a single carboxylate was discovered as HNF4α ligand, although the nuclear receptor is known to bind fatty acids [4] and the screening library comprised 47 (10%) carboxylic acid-containing fragments. In line with the high constitutive transcriptional inducer activity of HNF4α, we observed bidirectional modulation of the nuclear receptor in the screening Gal4–HNF4α assay and in the native cellular setting in HepG2 cells. The active fragments exhibited different types of activity including agonism and inverse agonism, and direct interaction with the HNF4α LBD was confirmed for **7**, **9**, and **10**. The most active compounds **7**, **9**, and **10**, therefore, present as appealing starting points for medicinal chemistry as they offer favorable ligand efficiencies, low molecular weights, and low lipophilicity. Their scaffolds are simple and common, allowing rapid diversification and structure–activity relationship studies.

The ligand-activated transcription factor HNF4α has been found to involve in metabolic balance and cancer development, but, to date, knowledge on its ligands is very limited. Potent modulators for the nuclear receptor are urgently needed to validate its promising therapeutic potential in metabolic diseases and oncology. We disclose three orthogonally validated HNF4α ligands (**7**, **9**, and **10**) as high-quality chemical starting matter for medicinal chemistry. Additionally, our results provide further evidence that activity of the poorly studied nuclear receptor HNF4α can be controlled by small-molecule ligands in a bidirectional fashion thus offering great potential as a molecular drug target. Modulatory effects of **4**, **6**, **7**, **9**, and **10** on expression levels of the HNF4α-regulated gene FBP1 in a native cellular setting indicate that this set of HNF4α modulators can also serve as an initial chemogenomic tool for early functional and phenotypic studies. Still, further efforts are needed to develop highly optimized HNF4α ligands as probes to question the therapeutic potential of the nuclear receptor in depth.

## 4. Materials and Methods

### 4.1. Hybrid Gal4-HNF4α Reporter Gene Assay

*Plasmids*: The Gal4-fusion receptor plasmid pFA-CMV-hHNF4α-LBD coding for the hinge region and LBD of the canonical isoform of HNF4α (uniprot entry: P41235-1, residues 142-377) was constructed by integrating cDNA fragments obtained from PCR amplification using natural cDNA (purchased as cDNA clone IRCBp5005M2212Q from Source BioScience, Nottingham, UK) as template between the BamHI cleavage site of the pFA-CMV vector (Stratagene, La Jolla, CA, USA) and an afore inserted KpnI cleavage site as described previously [22,23,24]. The frame and sequence of the fusion plasmid were verified by sequencing. pFR-Luc (Stratagene) was used as reporter plasmid and pRL-SV40 (Promega, Madison, WI, USA) served for normalization of transfection efficiency and cell growth. The Gal4-VP16 [25] expressed from plasmid pECE-SV40-Gal4-VP16 [26] (Addgene, entry 71728, Watertown, MA, USA) was used as a ligand-independent transcriptional inducer for control experiments. *Assay Procedure*: HEK293T cells were grown in Dulbecco’s Modified Eagle Medium (DMEM) high glucose, supplemented with 10% Fetal Bovine Serum (FCS), sodium pyruvate (1 mM), penicillin (100 U/mL), and streptomycin (100 μg/mL) at 37 °C and 5% CO_2_. The day before transfection, HEK293T cells were seeded in 96-well plates (3.0·10^4^ cells/well). Before transfection, the medium was changed to Opti-MEM without supplements. Transient transfection was carried out using Lipofectamine LTX reagent (Invitrogen, Carlsbad, CA, USA) according to the manufacturer’s protocol with pFR-Luc (Stratagene), pRL-SV40 (Promega), and the pFA-CMV-hHNF4α-LBD clone or pECE-SV40-Gal4-VP16 for control experiments. Five hours after transfection, the medium was changed to Opti-MEM supplemented with penicillin (100 U/mL), streptomycin (100 μg/mL), now additionally containing 0.1% DMSO (0.2% in the primary screen) and the respective test compounds or 0.1% DMSO (0.2% in the primary screen) alone as the untreated control. In the primary screening, the core set of the Prestwick Drug Fragment Library (Prestwick Chemical, Illkirch, France) was tested at 50 µM concentration in two biologically independent repeats. In the follow-up and dose-response profiling, each concentration was tested in duplicates in at least three biologically independent repeats. Following overnight (12−14 h) incubation with the test compounds, cells were assayed for luciferase activity using Dual-Glo™ luciferase assay system (Promega) according to the manufacturer’s protocol. Luminescence was measured with a Tecan Spark 10M luminometer (Tecan Group Ltd., Männedorf, Switzerland). Normalization of transfection efficiency and cell growth was done by division of firefly luciferase data by renilla luciferase data and multiplying the value by 1000, resulting in relative light units (RLU). Fold activation was obtained by dividing the mean RLU of test compounds at a respective concentration by the mean RLU of untreated control.

### 4.2. Recombinant HNF4α LBD Expression and Purification

The LDB domain of HNF4α (aa. 148–377) subcloned into pNIC28-Bsa4 was expressed in *E. coli* Rosetta. The recombinant protein harboring an N-terminal His_6_ tag was initially purified by Ni^2+^-affinity chromatography. Size exclusion chromatography was performed using Superdex S75 column (GE Healthcare, Chicago, IL, USA.), and the pure protein was stored in a buffer containing 20 mM 4-(2-hydroxyethyl)-1-piperazineethanesulfonic acid (HEPES) pH 7.5, 200 mM NaCl, 0.5 mM tris(2-carboxyethyl)phosphine (TCEP), and 5% *w*/*v* glycerol.

### 4.3. Quantification of Human FBP1 mRNA Expression in HepG2 Cells by Quantitative Real-Time Polymerase Chain Reaction (qRT-PCR)

HepG2 cells were grown in 12-well plates in DMEM high glucose, supplemented with 10% FCS, sodium pyruvate (1 mM), penicillin (100 U/mL), and streptomycin (100 μg/mL) at 37 °C and 5% CO_2_ to approximately 60% confluence. Before incubation with test compounds, cells were kept in MEM supplemented with 1% charcoal-stripped FCS, penicillin (100 U/mL), and streptomycin (100 μg/mL) for 24 h [27]. For gene expression analysis, cells were incubated with **4** (50 μM), **6** (10 μM), **7** (50 μM), **9** (50 µM), **10** (50 µM), or 0.1% DMSO as untreated control in the same medium for 8 h. The cells were then harvested and directly used for RNA extraction by the E.Z.N.A. Total RNA Kit I (R6834-02, Omega Bio-Tek, Inc., Norcross, GA, USA). Three micrograms of total RNA extracted from HepG2 cells were reverse-transcribed into cDNA using the High-Capacity RNA-to-cDNA Kit (4387406, Thermo Fischer Scientific, Inc., Waltham, MA, USA) according to the manufacturer’s protocol. HNF4α-regulated FBP1 expression was evaluated by qRT-PCR analysis with a StepOnePlus System (Life Technologies, Carlsbad, CA, USA) using Power SYBR Green (Life Technologies; 12.5 μL/well). Each sample was set up in duplicates and repeated in three independent experiments. Data were analyzed by the comparative 2^−ΔΔCt^ method with glyceraldehyde 3-phosphate dehydrogenase (GAPDH) as the reference gene. The following PCR primers were used: hGAPDH [28]: 5′-ATA TGA TTC CAC CCA TGG CA (fw), 5′-GAT GAT GAC CCT TTT GGC TC (rev) and hFBP1 [19]: 5′-AGC CTT CTG AGA AGG ATG CTC (fw), 5′-GTC CAG CAT GAA GCA GTT GAC (rev).

### 4.4. Isothermal Titration Calorimetry

Isothermal titration calorimetry was performed on an Affinity ITC (TA Instruments, New Castle, DE, USA). The instrument was equilibrated to 25 °C and the stirring rate was set to 75 rpm. HNF4α buffer (20 mM HEPES pH 7.5, 200 mM NaCl, 0.5 mM TCEP, 5% *w*/*v* glycerol) with up to 1% DMSO (final concentration) was used for ITC. The recombinant HNF4α LBD protein (10–20 µM, 172 µL) was titrated with fragments (**4**, **6**, **7**, **9**, **10**; 50–200 µM; dissolved in the same buffer). A total of 20-30 injections with a volume of 2.5–4 µL and with an interval of 200–300 s were performed. As a control experiment, HNF4α protein was titrated with buffer, and the buffer was titrated with test compound to detect dissolution artifacts with otherwise identical experimental parameters. Data analysis was performed with NanoAnalyze^TM^ Software (TA Instruments, New Castle, DE, USA.) using an independent binding model.

## Figures and Tables

**Table 1:** Primary screening hits with primary screening data and control experiment on VP16 for non-specific activity. Primary screening data are the mean of two biologically independent repeats. HNF4α follow-up data and VP16 control data are the mean ± SD fold activation of reporter activity of at least four biologically independent repeats in duplicates. n.s.—not significant (*p* ≥ 0.05), ** *p* < 0.01, *** *p* < 0.001 (*t*-test).

ID	Structure	Primary Screen (Fold Act., 50 µM)	Follow-up HNF4α	VP16 Control	HNF4α vs. VP16
**1**	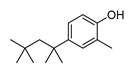	2.48	1.1 ± 0.2 (50 µM)	0.80 ± 0.07 (50 µM)	n.s.
**2**	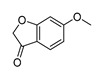	1.55	1.3 ± 0.2 (50 µM)	1.3 ± 0.3 (50 µM)	n.s.
**3**	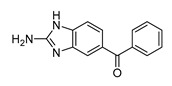	2.09	1.5 ± 0.2 (50 µM)	1.7 ± 0.3 (50 µM)	n.s.
**4**	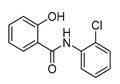	5.18	4 ± 2 (50 µM)	0.5 ± 0.2 (50 µM)	*p* = 0.0014 (**)
**5**	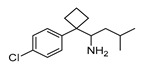	1.80	2.5 ± 0.2 (100 µM)	1.1 ± 0.1 (100 µM)	*p* < 0.0001 (***)
**6**	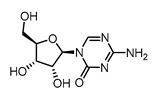	5.48	2.4 ± 0.5 (5 µM)	1.1 ± 0.1 (5 µM)	*p* = 0.0034 (**)
**7**	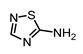	2.36	3.0 ± 0.3 (50 µM)	1.4 ± 0.1 (50 µM)	*p* < 0.0001 (***)
**8**	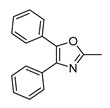	2.09	1.1 ± 0.2 (50 µM)	0.92 ± 0.07 (50 µM)	n.s.
**9**	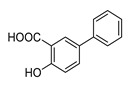	0.54	0.6 ± 0.1 (30 µM)	1.2 ± 0.2 (30 µM)	*p* < 0.0001 (***)
**10**	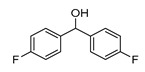	0.63	0.75 ± 0.05 (50 µM)	1.1 ± 0.2 (50 µM)	*p* = 0.0057 (**)
**11**	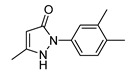	0.32	0.21 ± 0.03 (100 µM)	0.21 ± 0.02 (100 µM)	n.s.

**Table 2:** Validated HNF4α ligands with control experiment HNF4α modulatory activity and binding affinity to the recombinant HNF4α ligand-binding domain (LBD). EC_50_ and IC_50_ values were calculated from dose–response curves and are the mean ± SD of at least four biologically independent repeats in duplicates. Fold and remaining (rem.) activation (act.) refer to the maximum fold increase or decrease in reporter activity relative to 0.1% DMSO. Binding was determined by isothermal titration calorimetry (ITC). n.d.—not determined

ID	Structure	HNF4α Ligand Type	Validated HNF4α Modulation	HNF4α LBD Binding
**4**	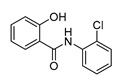	Agonist	EC_50_ 15 ± 1 µM 5.6 ± 0.3-fold act.	no/weak binding
**5**	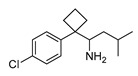	Agonist	EC_50_ > 100 µM	n.d.
**6**	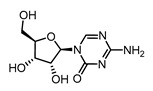	Agonist	EC_50_ 5.8 ± 0.6 µM 6.1 ± 0.7-fold act.	no binding
**7**	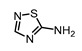	Agonist	EC_50_ 31 ± 8 µM 3.8 ± 0.6-fold act.	K_d_ 7 µM
**9**	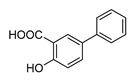	Inverse agonist	IC_50_ 8 ± 2 µM 0.57 ± 0.04 rem. act.	K_d_ 0.3 µM
**10**	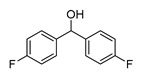	Inverse agonist	IC_50_ 24 ± 5 µM 0.65 ± 0.05 rem. act.	K_d_ 1.7 µM

**Table 3:** Calculated lipophilicity and ligand efficiency metrics of HNF4α modulators. LogP and logS were retrieved from the ALOGPS 2.1 resource [20]. Ligand efficiency (LE), lipophilic ligand efficiency (LLE), and size-independent ligand efficiency (SILE) were calculated as described in Reference [21].

ID	HNF4α Activity	LogP	LogS	LE	LLE	SILE
**4**	pEC_50_ 4.8	4.13	−3.72	0.39	0.69	2.1
**5**	pEC_50_ < 4	4.84	−5.50	<0.32	<0	<1.7
**6**	pEC_50_ 5.2	−2.45	−1.30	0.42	7.7	2.2
**7**	pEC_50_ 4.5	−0.19	−0.95	1.03	4.7	2.6
**9**	pIC_50_ 5.1	3.77	−2.85	0.44	1.3	2.2
**10**	pIC_50_ 4.6	3.01	−3.65	0.40	1.6	2.0

## Supplementary Materials

Supplementary materials can be found at https://www.mdpi.com/1422-0067/21/21/7895/s1. Table S1. All molecular structures of library compounds with associated primary screening data.

## Abbreviations

FBP1	fructose-1,6-bisphosphatase 1
HNF4α	hepatocyte nuclear factor 4α
LBD	Ligand-binding domain
MODY	maturity-onset diabetes of the young
RLU	relative light units
